# Clinical responses to vemurafenib in postoperative recurrence of papillary thyroid carcinoma with esophageal fistula: A case report

**DOI:** 10.1097/MD.0000000000037513

**Published:** 2024-03-15

**Authors:** Sicheng He, Wei Lu, Xun Ding, Jun Zhou, Di Liu, Yang Zhu, Fugang Yang, Zanmei Fu

**Affiliations:** a The Interventional Diagnostic and therapeutic Center, Zhongnan Hospital of Wuhan University, Wuhan, China.

**Keywords:** BRAF^V600E^, Esophageal fistula, lenvatinib, next-generation sequence (NGS), papillary thyroid cancer, postoperative recurrence, vemurafenib

## Abstract

**Background:**

While papillary thyroid carcinoma (PTC) generally exhibits a favorable prognosis post-surgery, the poorly differentiated subtype presents elevated rates of postoperative recurrence. Certain aggressive cases demonstrate invasive behavior, compromising adjacent structures and leading to a poor prognosis. This study delineates a unique case of postoperative PTC recurrence, complicated by esophageal fistula, that showed favorable outcomes following brief Vemurafenib treatment.

**Patient description:**

A 64-year-old female patient underwent surgical resection for PTC, subsequently experiencing rapid tumor recurrence and development of an esophageal fistula.

**Diagnosis:**

The patient was confirmed to have locally advanced PTC through intraoperative cytopathology. The cancer recurred postoperatively, culminating in the formation of an esophageal fistula.

**Methods:**

The patient was administered Vemurafenib at a dosage of 960 mg twice daily following tumor recurrence.

**Results:**

A 12-month regimen of targeted Vemurafenib therapy led to a substantial reduction in tumor size. Concurrently, the esophageal fistula underwent complete healing, facilitating successful removal of the gastrostomy tube. The tumor response was classified as stable disease.

**Conclusion subsections:**

Vemurafenib demonstrates potential as a targeted therapeutic strategy for recurrent PTC harboring the BRAF^V600E^ mutation. This approach may effectively mitigate tumor dimensions and the associated risk of esophageal and tracheal fistulas.

## 1. Introduction

Recent epidemiological studies underscore a marked increase in the incidence of thyroid cancer, with papillary thyroid carcinoma (PTC) constituting the predominant subtype and generally associated with favorable prognosis.^[1]^ Despite a 5-year mortality rate as low as 2%, a subset of PTC exhibits aggressive behavior with a propensity for metastasis or recurrence.^[2]^ Well-differentiated PTC often presents with a positive prognostic outlook. However, invasive forms of the disease are less amenable to standard treatments like iodine-131 therapy and thyroid stimulating hormone suppression, leading to elevated rates of postoperative recurrence. Further complicating the clinical picture are instances where metastatic lymph nodes infiltrate adjacent structures such as the esophagus and trachea, significantly impacting survival rates.^[3,4]^

For recurrent PTC, multiple invasive treatment options are extant, including radiation therapy (with the option of radioactive I125 seed implantation), systemic chemotherapy (ChT), immune checkpoint inhibitors (iCTs), molecularly targeted therapy, arterial chemoembolization (employing both thermal and cryoablation techniques), and ethanol ablation. Precise and timely treatment selection is critical, particularly for cases exhibiting postoperative recurrence.^[5]^ However, these conventional treatments occasionally yield a range of adverse effects, making them unsuitable for certain patients. In such instances, targeting specific gene mutations offers a viable therapeutic strategy.

The pathogenesis of PTC primarily involves genetic mutations or aberrations during cellular growth. Agents targeting vascular endothelial growth factor receptors (VEGFR) are instrumental in molecularly targeted treatments for PTC. Additionally, kinases within proto-oncogenes can initiate mutations that serve as effective therapeutic targets. Although point mutations in the RAS gene are pervasive across various forms of thyroid cancer, the frequency of RAS mutations in PTC is markedly lower than that of BRAF mutations.^[6]^ Notably, 45% of PTC cases harbor BRAF gene mutations, with the e600 codon comprising 90%, thus underscoring the significance of molecularly targeted therapy.^[3]^ Vemurafenib, a BRAF^V600E^ kinase inhibitor, has demonstrated clinical efficacy against advanced PTC with BRAF mutations in Phase II trials conducted by MD Anderson Cancer Center.^[7]^ In this context, we present a case of a patient with postoperative recurrent PTC who required percutaneous gastrostomy due to esophageal invasion following localized progression of the tumor. After initiating Vemurafenib treatment, the tumor dimensions were substantially reduced, the esophageal fistula healed, and the gastrostomy tube was successfully removed without complications.

## 2. Case presentation

In February 2022, a 58-year-old female patient was admitted to our medical center, having previously undergone resection for right PTC 4 months prior at a local hospital. Postoperatively, she was treated with Lenvatinib and levothyroxine sodium. She presented to our department with a swollen right neck and elevated skin temperature (Fig. 5A). Her medical history was devoid of hypertension, diabetes, heart disease, hyperthyroidism, infectious diseases, autoimmune disorders, or familial illnesses. Upon comprehensive assessment, tumor recurrence was suspected.

**Figure 1.**
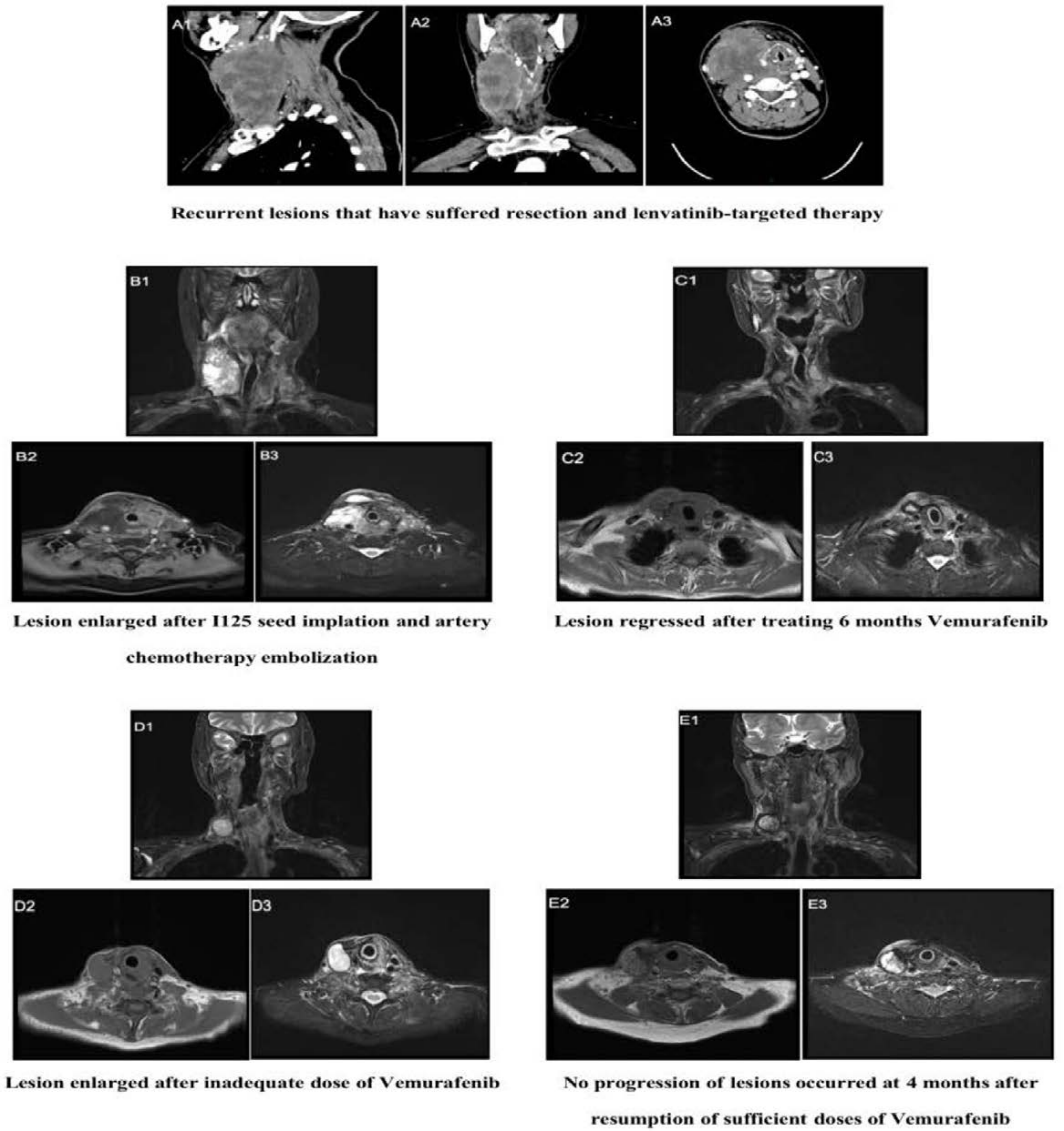
(A) Baseline CT scan of the thyroid conducted in February 2022; lesion dimensions were 45 × 58 × 101 mm. (B) Subsequent MRI post-I125 seed implantation and arterial chemoembolization in April 2022 demonstrated a lesion measuring 42 × 35 × 72 mm. (C) MRI evaluation after 6 months of vemurafenib therapy in November 2023 revealed significant tumor reduction to dimensions of 8 × 4 × 2 mm. (D) MRI obtained in February 2023 post-limited vemurafenib administration indicated lesion dimensions of 29 × 21 × 25 mm. (E) MRI conducted in June 2023, following the reinitiation of optimal vemurafenib dosing, indicated further tumor response.

**Figure 2.**
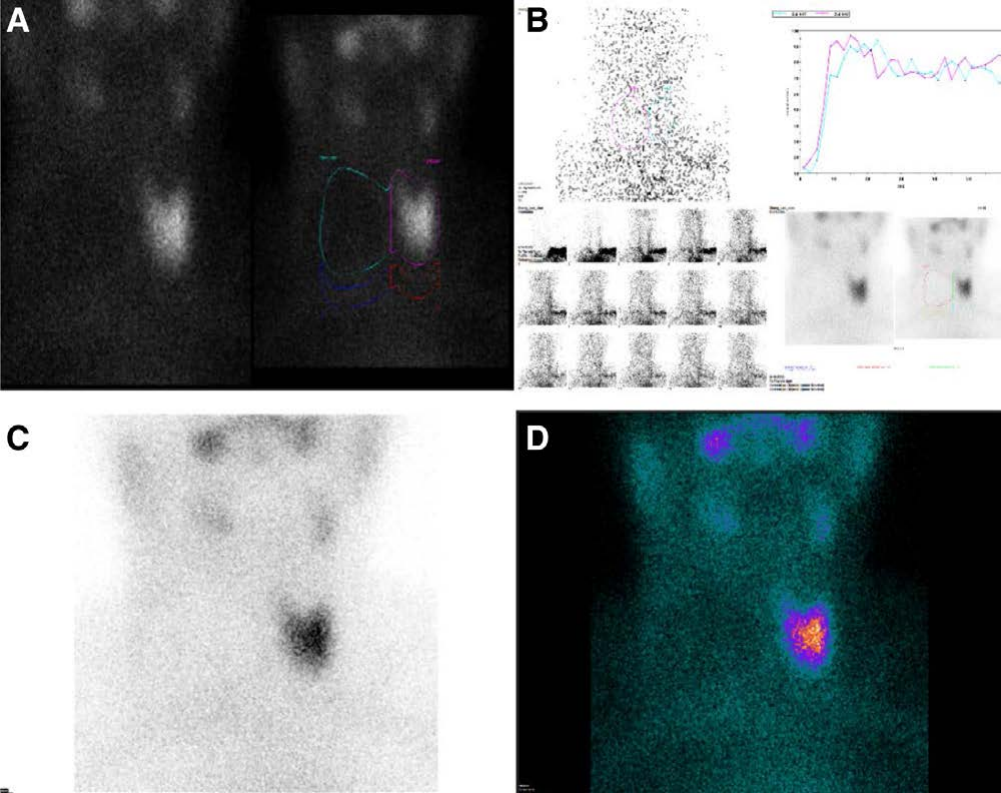
(A–D) Thyroid scintigraphy in February 2022: Parameters include thyroid technetium uptake at 0.5% and thyroid-to-background ratio (T/B) at 1.7. The lack of technetium uptake in the right lobe and isthmus denotes a “cold nodule,” accompanied by diminished global ^99m^TcO4^−^ uptake in the thyroid.

**Figure 3.**
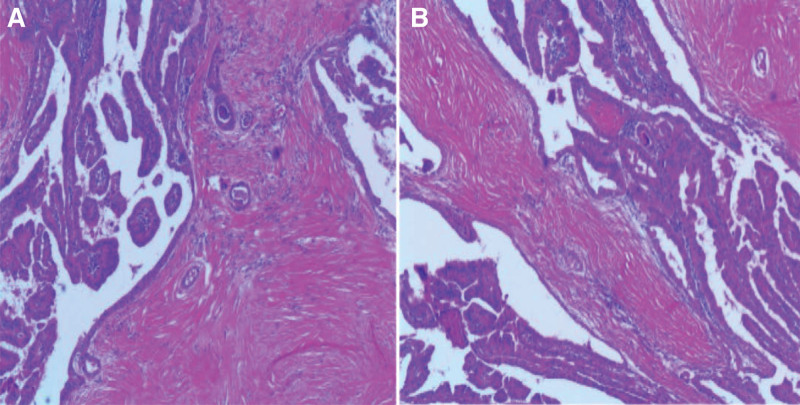
(A–B) Hematoxylin and Eosin (H&E) stained sections from left thyroidectomy illustrate the presence of classical papillary thyroid carcinoma.

**Figure 4.**
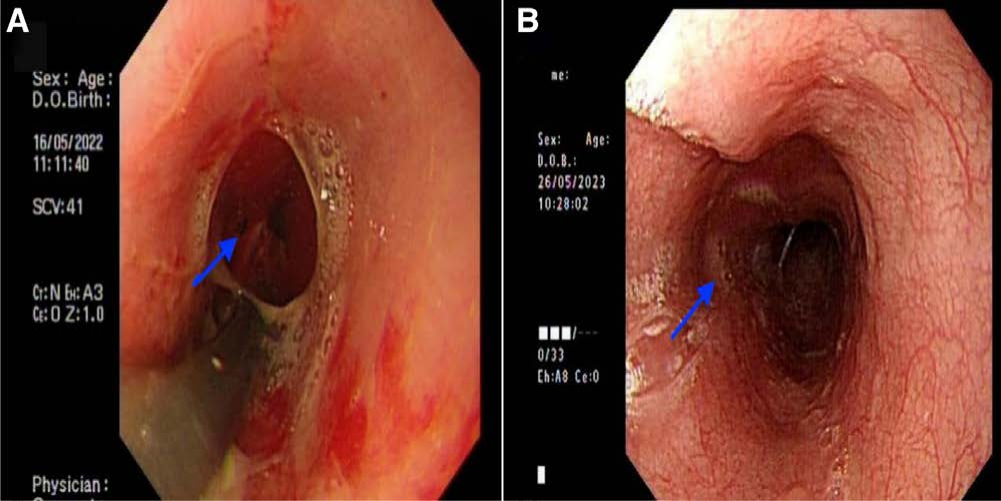
(A–B) Depicts the status of the esophageal fistula prior to and following a 12-month treatment regimen with Vemurafenib.

**Figure 5.**
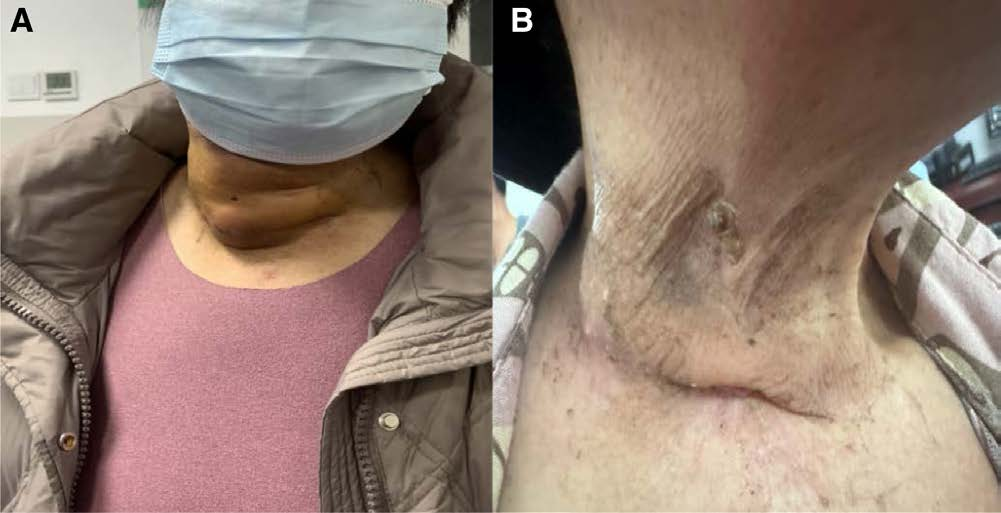
Reveals observable alterations in the right neck lesion pre-and post-therapeutic intervention over an 18-month period.

Contrast-enhanced computed tomography scans revealed soft tissue invasion in the neck, encasement of the right cervical artery, and invasion of the right internal jugular vein. Multiple lymph node metastases were identified in bilateral cervical zones V, VI, VII, as well as the supraclavicular area, thoracic inlet, trachea, and periesophagus (Fig. 1A). ECT thyroid scintigraphy demonstrated that the right lobe and isthmus of the thyroid were “cold nodules,” lacking technetium uptake (Figs. 2A–D). The multi-disciplinary team concluded that the patient’s esophageal and tracheal structures were compromised, diagnosing the condition as PTC T4bN1bM0 (Stage IVa). Secondary resection was deemed unfeasible, and I131 therapy was contraindicated due to lack of technetium absorption. Lenvatinib, a small molecule tyrosine kinase inhibitor, was discontinued due to disease progression. Subsequently, metastatic arterial chemoembolization and I125 seed implantation were implemented to halt tumor growth. Despite undergoing 3 cycles of Epirubicin + Cisplatin for arterial chemoembolization and I125 seed implantation, there was negligible reduction in the size of metastatic lymph nodes in the patient’s right neck, and the disease progressed, invading the esophagus. This indicated the inefficacy of the current treatment regimen in controlling lesion progression. Magnetic Resonance Imaging further revealed poor demarcation of the mass from adjacent structures, including the right submandibular gland, hypopharynx, esophagus, trachea, and larynx, with the trachea and esophagus displaced laterally due to compression (Fig. 1B).

Compounding the patient’s deteriorating condition, an esophageal fistula developed subsequent to lesion progression, as evidenced by digestive endoscopy (Fig. 4A). Surgical repair was deemed unfeasible following a meticulous evaluation. In light of her inability to ingest food via the esophagus, percutaneous gastrostomy was performed to maintain nutritional intake. Given the aggressive nature of the tumor, invasive procedures were considered contraindicated.

Upon reevaluation of the pathological specimens from the initial right thyroidectomy, the diagnosis of classic PTC was reaffirmed (Fig. 3A, B). In pursuit of effective therapeutic strategies, Next-Generation Sequencing was performed on prior biopsy samples, utilizing a cancer-specific gene panel (Table 1). The results revealed a p.V600E exon 15 missense mutation at the BRAF locus, characterized by c.1799T > A, with an allele fraction (AF) of 15% in the tissue and 0.4% in the plasma (Table 1). Given the elevated tissue AF, the utilization of BRAF inhibitors was deemed a viable option. Encouraged by the proven efficacy of BRAF inhibitors in melanoma, the multi-disciplinary team unanimously endorsed Vemurafenib as the chosen therapeutic agent, to be administered at a dosage of 960 mg twice daily.

**Table 1:** Next-generation sequence (NGS).

Tumor-specific mutation				
Gene	Mutation	Mutant	Plasma abundance	Tissue abundance
BRAF	p.V600E exon 15 missense mutation	c.1799T > A (p.V600E)	0.40%	15.00%
TP53	p.M246I exon 7 missense mutation	c.738G > A (p.M246I)	–	1.40%
TERT	c.-124C > T promoter mutation	c.-124C > T	–	13.60%
ATM	p.P2699R exon 55 missense mutation	c.8096C > G (p.P2699R)	—	3.10%
NF1	p.D832H exon 21 missense mutation	c.2494G > C (p.D832H)	–	3.90%
TAP1	p.N311T exon 3 missense mutation	c.932A > C (p.N311T)	–	4.30%

Following a 6-month treatment regimen with Vemurafenib, the patient exhibited a significant reduction in right neck metastatic lymph nodes, achieving a partial response (PR) with approximately 90% tumor shrinkage (Fig. 1C). Throughout this period, the patient remained dependent on a gastrostomy tube for nutritional intake, resulting in a 5 kg weight loss. Notably, Vemurafenib administration was devoid of adverse effects, thereby affirming its clinical safety and tolerability. However, resumption of oral feeding remained unfeasible due to incomplete healing of the esophageal fistula. Subsequently, the patient reduced the Vemurafenib dose to 480 mg twice daily for economic considerations, leading to the reenlargement of the right neck metastatic lymph nodes (Fig. 1D). It remains inconclusive whether this tumor progression was attributable to suboptimal dosing or drug tolerance. The patient achieved a progress-free survival (PFS) of 10 months. After 12 months of Vemurafenib treatment, digestive endoscopy revealed an absence of esophageal abnormalities (Fig. 4B). The fistula’s closure was primarily attributed to the inhibitory effects of Vemurafenib on tumor progression and secondarily to esophageal disuse, fostering fibrous connective tissue hyperplasia that facilitated fistula repair. Consequently, the gastrostomy tube was successfully removed, and the patient regained oral feeding capabilities. To manage disease progression, the patient was prescribed 100 µg of Levothyroxine sodium tablets to be taken orally once daily (QD), in conjunction with an adequate Vemurafenib dosage (960 mg twice daily). A follow-up examination at 4 months indicated an absence of significant neck skin swelling (Fig. 5B), and no further extension of right cervical metastatic lymph nodes was observed (Fig. 1E). According to mRECIST criteria, the tumor response was classified as stable disease, likely attributable to the reinstatement of adequate Vemurafenib dosing. Future efforts will focus on establishing a long-term follow-up protocol to monitor and document the patient’s ongoing recovery status.

## 3. Discussion

Management of recurrent, unresectable metastatic PTC remains an unresolved challenge due to the absence of targeted and effective therapeutic strategies. The 8th edition of the American Joint Committee on Cancer Cancer Staging Manual has undergone significant revisions in thyroid cancer staging, including an adjustment in the age parameter for poor prognosis from 45 to 55 years. For early-stage tumors (Stages I and II), nodal involvement serves as the principal determinant in staging. In advanced tumors, the assessment of extrathyroidal extension (ETE) becomes pivotal, particularly concerning invasion into adjacent neck structures such as the esophagus, trachea, and nerves.^[8]^ Early-stage PTC benefits from a multifaceted treatment approach encompassing partial or total thyroidectomy, lymph node dissection, radioactive iodine ablation, and suppression of thyroid stimulating hormone. This approach has yielded a 10-year survival rate exceeding 85%. However, late-stage patients with ETE have a survival rate <10%.^[9]^ BRAF protein plays a significant role in cancer therapeutics as a component of the RAF-MEK-ERK signaling pathway. It influences cellular metabolism, proliferation, and apoptosis. The most prevalent form of BRAF mutation in metastatic PTC is BRAF^V600E^, which is implicated in tumor differentiation, migration, and apoptosis.^[10]^ Specifically, the T1799A transversion mutation is integral to BRAF^V600E^ and activates the downstream MAPK signaling pathway, thereby imparting oncogenic properties to the kinase by modulating the V600E amino acids in the BRAF protein.^[11]^ This mutation fosters activation of BRAF, MEK, and ERK1/2 kinases, subsequently elevating the risk of postoperative recurrence and mortality.^[12]^ The prognosis for our patients harboring the T1799A BRAF gene mutation is notably poor, underscoring the critical need for tailored therapeutic interventions in this patient population.

Vemurafenib (PLX4032) acts as an inhibitor for the serine-threonine kinase BRAF mutant family. It effectively mitigates cellular proliferation by binding to the ATP active site of the BRAF^V600E^ kinase. This compound exhibits specific affinity for mutant BRAF, thereby disrupting the BRAF/MEK interaction within the BRAF/MEK/ERK signaling pathway and subsequently inducing apoptosis.^[13]^ Dabrafenib serves as another potent ATP-competitive inhibitor, yielding significant therapeutic benefits in various BRAF^V600E^-mutated cancers including melanoma, advanced non-small-cell lung cancer, and anaplastic thyroid cancer.^[10]^ Evidence from Phase I clinical trials suggests that Dabrafenib is both well-tolerated and efficacious in patients with BRAF^V600E^ mutant thyroid cancer, showing a median progression-free survival (mPFS) of 11.3 months, albeit within a limited sample size.^[14]^ Another Phase I study focusing on Vemurafenib demonstrated PR and stable treatment outcomes in patients who had recurrent PTC following thyroidectomy or radioactive iodine ablation. The overall survival duration for these patients ranged between 15 and 31.7 months.^[3]^ Moreover, a multicenter, open-label Phase II trial revealed that 38.5% of patients, who had not previously been treated with VEGFR multikinase inhibitors, achieved PR. The rate of stable disease was 57.7%, while disease progression (PD) was observed in only 3.8% of the cases.^[15]^ These clinical trials underscore the pivotal role of Vemurafenib in the targeted treatment of recurrent PTC, warranting further investigation to substantiate its therapeutic efficacy.

Prior studies have extensively examined the effectiveness of antiangiogenic tyrosine kinase inhibitors such as Sorafenib and Lenvatinib in targeted treatment. Both inhibitors act on a similar set of targets, including VEGFR, FGFR, PDGFR, RET, and KIT, although Sorafenib is a less potent inhibitor of BRAF. In a Phase II single-arm trial involving Sorafenib, the PR rate among patients with PTC was 13%, and PFS reached 15 months. The rate of disease progression (PD) was documented at 13%.^[16]^ Lenvatinib exhibited promising outcomes in a preliminary trial on radioactive iodine-refractory thyroid cancer; PFS was 18.3 months, and the overall response rate (comprising CR + PR) was 64.8%. The data also indicated significant efficacy in treating bone metastases.^[16]^ To date, no prospective studies have directly compared the effectiveness of Vemurafenib with Sorafenib or Lenvatinib. However, in cases of PTC with BRAF^V600E^ mutations, patients unresponsive to first-line treatments with Lenvatinib or Sorafenib may derive benefit from Vemurafenib.^[17]^ In our study, switching from first-line Lenvatinib to Vemurafenib resulted in successful disease control. Emerging research suggests that other TKIs such as Vandetanib, which inhibits EGFR/VEGFR/RET, or Sunitinib, which inhibits PDGFRβ/VEGFR/FGFR, could offer further therapeutic advantages in PTC patients.^[18]^ Given that resistance to BRAF inhibitor monotherapy may arise from the reactivation of the MAPK pathway due to upregulation of EGFR/PDGFRβ, the combination of Vandetanib or Sunitinib with Vemurafenib might present a future therapeutic avenue for patients with BRAF^V600E^-positive PTC.^[18]^

While Vemurafenib demonstrates notable efficacy in the management of unresectable late-stage recurrent PTC, a comprehensive evaluation must consider its toxicological profile. Adverse effects, as delineated in Phase II clinical trials of Vemurafenib, primarily encompass dermatological manifestations, altered cognitive states, anorexia, weight variations, anosmia, and alopecia.^[15]^ In our cohort, patients exhibited minimal severe complications post-Vemurafenib administration; fatigue was the predominant observed adverse event. This symptomatology likely stems from insufficient nutritional absorption, given the patient’s reliance on enteral feeding via a gastrostomy tube.

To summarize, this case report serves as the inaugural documentation of Vemurafenib utility in managing postoperative recurrence BRAF^V600E^-positive PTC accompanied by esophageal fistula. Despite the utilization of various therapeutic strategies, Vemurafenib effectively inhibited metastatic lymph node growth and achieved significant tumor reduction. Crucially, the treatment resulted in the complete healing of the esophageal fistula, restoring normal feeding function. These outcomes furnish novel therapeutic options for inoperable cases presenting with tracheal or esophageal fistula, extending potentially to those previously administered multikinase inhibitors. Nonetheless, future investigations, encompassing randomized controlled trials and studies focused on Vemurafenib resistance mechanisms, are requisite for the definitive assessment of its therapeutic efficacy and safety profile.

## Acknowledgments

We thank the patient in this report and her family. Written informed consent was obtained from the patient for publication of this case report and any accompanying images.

## Author contributions

Writing – original draft: Sicheng He.

Writing – review & editing: Sicheng He.

Supervision: Wei Lu.

Resources: Sicheng He, Xun Ding, Jun Zhou.

Visualization: Sicheng He, Fugang Yang.

Conceptualization: Sicheng He, Yang Zhu.

Data curation: Sicheng He, Di Liu.

Formal analysis: Sicheng He, Zanmei Fu.
